# Impacts of MicroRNAs Induced by the Gut Microbiome on Regulating the Development of Colorectal Cancer

**DOI:** 10.3389/fcimb.2022.804689

**Published:** 2022-04-14

**Authors:** Juan Xing, Yiqun Liao, Huan Zhang, Wenjie Zhang, Zhilin Zhang, Jie Zhang, Daorong Wang, Dong Tang

**Affiliations:** ^1^ Clinical Medical College, Yangzhou University, Yangzhou, China; ^2^ Department of Clinical Medical College, Dalian Medical University, Dalian, China; ^3^ Department of General Surgery, Institute of General Surgery, Clinical Medical College, Yangzhou University, Yangzhou, China

**Keywords:** colorectal cancer, microRNAs, gut microbiome, bioactive dietary components, modulation

## Abstract

Although a dysfunctional gut microbiome is strongly linked to colorectal cancer (CRC), our knowledge of the mediators between CRC and the microbiome is limited. MicroRNAs (miRNAs) affect critical cellular processes, such as apoptosis, proliferation, and differentiation, and contribute to the regulation of CRC progression. Increasingly, studies found that miRNAs can significantly mediate bidirectional interactions between the host and the microbiome. Notably, miRNA expression is regulated by the gut microbiome, which subsequently affects the host transcriptome, thereby influencing the development of CRC. This study typically focuses on the specific functions of the microbiome in CRC and their effect on CRC-related miRNA production and reviews the role of several bacteria on miRNA, including *Fusobacterium nucleatum*, *Escherichia coli*, enterotoxigenic *Bacteroides fragilis*, and *Faecalibacterium prausnitzii*. Based on the important roles of miRNAs and the gut microbiome in CRC, strategies for modulating miRNA expression and regulating the gut microbiome composition need to be applied, such as bioactive dietary components and fecal microorganism transplantation.

## Introduction

The intestinal microenvironment averagely hosts more than 100 trillion bacteria, known as the gut microbiome. A healthy microbiome contributes to the maintenance of colonic microenvironment homeostasis, immune system development, and intestinal epithelial function (Yuan et al., 2018). When the composition and function of the microbiome are affected, diseases will occur accordingly, including colorectal cancer (CRC) (Wang et al., 2012). Although a dysfunctional gut microbiome is strongly linked to CRC, our knowledge of the mediators between CRC and the microbiome is limited.

In recent years, microRNAs (miRNAs) have increasingly caught the eye of scientists because of their important roles in the development and treatment of CRC. miRNAs are 20- to 22-nucleotide-long noncoding single-stranded RNAs with a highly stable structure (Bartel, 2004). In mammalian cells, miRNAs act as gene regulatory elements through posttranscriptional modifications *via* binding to target mRNAs (Fabian and Sonenberg, 2012). miRNAs regulate approximately 90% of the genes encoding proteins and affect critical cellular processes, such as apoptosis, proliferation, and differentiation. Its deregulation has also been implicated in tumor pathobiology, such as angiogenesis, immune surveillance, invasion, and metastasis (Chen et al., 2015; Chen et al., 2016). Studies have found that intestinal profiles of miRNA expression were differently expressed in the colon of colonized mice relative to germ-free mice (Dalmasso et al., 2011). This finding suggested that the microbiome can affect the expression of miRNAs, which in turn target its downstream genes and activate a new pathway, resulting in an influence on intestinal epithelial cells. However, because of the poor knowledge about the interactions between numerous unique miRNAs and the microbiome, it is challenging to study all possible pairwise interactions. Nevertheless, potential connections between unique miRNAs and the microbiome identified in CRC patients can be considered candidates for functional inspection. In this review, we present our understanding of the role of miRNAs in mediating CRC, thereby providing an idea that we can turn to diet regulation to treat and prevent CRC.

## Cancer-Related miRNAs and Their Interactions With the Microbiome and Host in CRC

**Figure 1:**
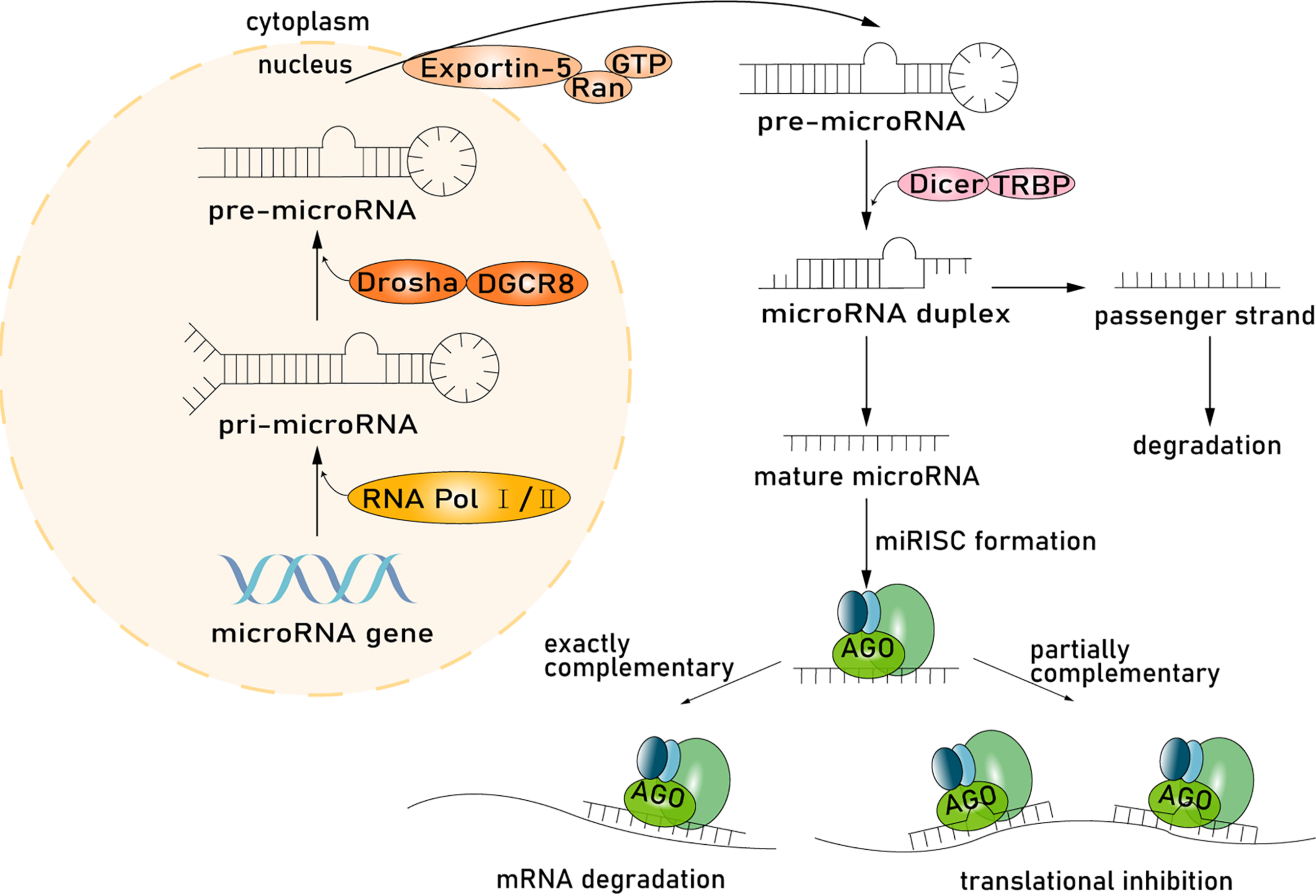
miRNA processing pathway. Host-derived miRNAs are synthesized in the nucleus and processed and then function in the cytoplasm. miRNA genes are transcribed into pri-miRNA through RNA polymerase II or RNA polymerase III subsequently cleaved by the microprocessor complex Drosha-DGCR8. The resulting precursor hairpins, the pre-miRNAs, are exported from the nucleus to the cytoplasm by exportin-5–Ran-GTP. In the cytoplasm, pre-miRNAs are cleaved into mature length by the Dicer in complex with TRBP. Functional strands of mature miRNAs are assembled with AGO proteins and a glycinetryptophan repeat-containing protein of 182 kDa (GW182), and then miRISC mediating target mRNAs silencing are recruited, whereas passenger strands are degraded. miRNAs regulate gene expression through two mechanisms. First, miRNA, with a wide range of complementary base pairs with mRNA, will guide miRISC to degrade mRNA, resulting in the instability or suppression of translation. Second, if miRNA and mRNA have partially complementary sequences, the miRISC will inhibit mRNA translation through the AGO protein.

Intestinal epithelial cells are the main producer of host-derived miRNAs. miRNAs are synthesized in the nucleus and processed and then function in the cytoplasm (Liu et al., 2016). miRNA genes are transcribed into primary miRNA transcripts (pri-miRNA) through RNA polymerase II or RNA polymerase III and are subsequently cleaved by the microprocessor complex Drosha–DGCR8 (Han et al., 2004; Lee et al., 2004; Borchert et al., 2006). The resulting precursor hairpins, the precursor miRNAs (pre-miRNAs), are exported from the nucleus to the cytoplasm by exportin-5–Ran-GTP (Yi et al., 2003). In the cytoplasm, pre-miRNAs are cleaved into mature length by the Dicer-TRBP complex. Functional strands of mature miRNAs are assembled with argonaute (AGO) proteins and a glycinetryptophan protein of 182 kDa (GW182), and then miRNA-induced silencing complexes (miRISC) mediating target mRNAs silencing are recruited, while passenger strands are degraded (Winter et al., 2009). miRNAs regulate gene expression, especially in mammalian cells, through two different albeit paired mechanisms. miRNAs have a wide range of complementary base pairs with mRNA and will guide the miRISC to the target mRNA and cause mRNA degradation, resulting in the instability or suppression of translation. Second, if the miRNA has partial complementary sequences to the 3′-untranslated region (3′-UTR) of the mRNA, the miRISC will inhibit mRNA translation through the AGO protein (Fabian and Sonenberg, 2012) (**Figure 1**). Many of these target mRNA transcripts play important roles in tumor proliferation, differentiation, and apoptosis (Winter and Diederichs, 2011), and studies have uncovered that each miRNA can target hundreds of mRNAs (Baek et al., 2008). Based on this vast and complex regulatory network, the regulatory function of miRNAs is immensely important in many signaling pathways, such as Wnt and APC, thereby influencing many aspects of tumor pathobiology in CRC (Li et al., 2016; Slattery et al., 2018).

In general, miRNA expression is strictly controlled in normal cells; however, defects in miRNA processing might occur in cancer cells, thereby enhancing tumorigenesis (Kumar et al., 2007). More and more data identified a large number of abnormal miRNA expression patterns in CRC, such as miR-21, miR-29, miR-34a, miR-124a, and miR-155 (Yi et al., 2016). These dysregulated miRNAs could be functionally delivered into the tumor microenvironment (TME) through tumor-derived exosomes) (Raposo and Stoorvogel, 2013). Since this seminal discovery, miRNAs’ ability to shape the complex inflammatory TME has emerged as a critical role in cell-to-cell communications. Studies demonstrated that miRNAs in the TME play critical roles in modulating the composition of the gut microbiome by regulating bacterial species, such as *Fusobacterium nucleatum* and *Escherichia coli*, thereby affecting gene regulation and growth effects (Liu et al., 2016). Similar regulation mediated by miRNAs is also found in stromal cells and immune cells (Bullock et al., 2013; Kohlhapp et al., 2015). In cancer cells, miRNAs encapsulated in microvesicles will be selectively transported to stromal cells and immune cells (Fanini and Fabbri, 2017), influencing their development, maturation, and antitumor activities (Fanini and Fabbri, 2017). A growing body of evidence has pointed to a central role of miRNAs in the dialogue between cancer and the immune system, with associated effects on the overall balance between immune-stimulation and immune escape (Kim et al., 2005; Keller et al., 2011; Fanini and Fabbri, 2017). In fact, the dysregulated oncogenic microbiome and immune system will create a more favorable TME for CRC cells. In addition to tumor-derived miRNAs, changes in the expression of miRNAs can also be attributed to an introduction of a foreign organism in the colon (Hu et al., 2015b). In the ileum of mice infected by *Listeria monocytogenes*, miR-378, miR-200c, miR-194, miR-200b, miR-148a, and miR-143 were usually downregulated. Meanwhile, miR-194 was downregulated, and miR-378 was upregulated in germ-free mice, with the rest having no influence (Archambaud et al., 2013). The abnormal expression of miRNAs subsequently activates the signaling pathways and regulates all aspects of tumor pathobiology in CRC (Li et al., 2014). Taken together, the bidirectional interaction between the host and microbiome mediated by miRNAs presents a new layer of complexity in the study of miRNAs.

## Microbial Regulation of CRC Mediated by miRNAs

### *F. nucleatum* Affects Cell Proliferation and Induces Chemoresistance in CRC Patients by Modulating miRNAs

**Figure 2:**
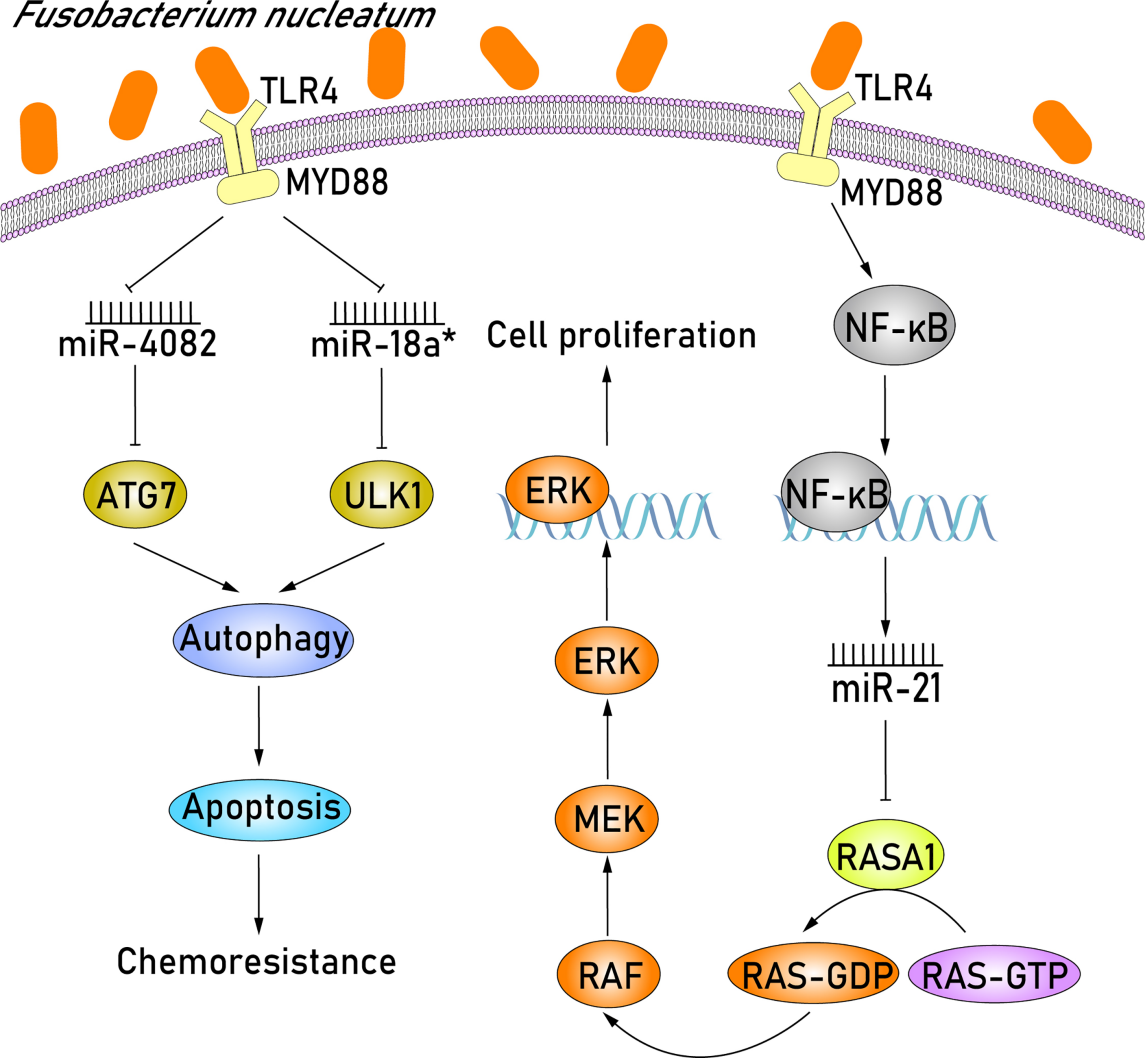
*F. nucleatum* promotes cell proliferation by upregulating miR-21 and induces chemoresistance in CRC patients by downregulating miR-4802 and miR-18a*. *F. nucleatum* initially recognizes TLR4/MYD88 signaling pathway and activates the key downstream effector NF-κB. NF-κB can bind to the promoter region of miR-21 and upregulate miR-21 expression, resulting in the subsequent downregulation of the downstream target gene RASA1. The partial functional loss of RASA1 can activate the GTPase activity of RAS, triggering the RAS-RAF-MEK-ERK (RAS-MAPK) signaling pathway. MAPK signaling pathway plays an important part in increasing cell proliferation, eventually leading to tumorigenesis. Furthermore, in the *F. nucleatum–*mediated chemoresistance of CRC cells, miR-4802 and miR-18a*, which are both dependent on the TLR4 and MYD88 signaling pathways, are significantly downregulated. The selective loss of miR-4802 and miR-18a* induces the upregulation of ATG7 and ULK1, respectively, which are members of autophagy signaling elements. Therefore, when these *F. nucleatum–*infected CRC cells are treated with chemotherapy agents, the autophagy pathway will be activated and consequently promote chemoresistance *via* suppressing cell apoptosis.

*F. nucleatum*, an anaerobic gram-negative bacterium, usually enriched in CRC and is closely related to colorectal carcinogenesis (Fukugaiti et al., 2015). Several recent studies have investigated the role of abnormal miRNA expression resulting from *F. nucleatum* infection in CRC development. Based on the miRNA expression profiles extracted from CRC tissues that were detected positive for *F. nucleatum* infection, Feng et al. (2019) demonstrated that miR-4474 and miR-4717 were significantly increased in early and advanced stage CRC. More importantly, the downregulation of CREB-binding protein (CREBBP) in CRC patients has also been studied and analyzed, which identified CREBBP as a novel target of miR-4474 and miR-4717. CREBBP, a transcriptional cofactor, is able to influence Wnt/β-catenin signaling and promote cell proliferation, likely affecting colonic tumorigenesis (Bordonaro and Lazarova, 2015). Thus, the overexpression of miR-4474 and miR-4717 in *F. nucleatum–*positive CRC tissues can inhibit cell tumor proliferation *via* degrading the mRNA of CREBBP. In addition to miR-4474 and miR-4717, miR-21 was also demonstrated to be dysregulated in *F. nucleatum*–positive CRC tissues (Shi et al., 2016; Yang et al., 2017) (**Figure 2**). It is widely acknowledged that *F. nucleatum* potentiates CRC development through Toll-like receptor 4 (TLR4) signaling, where TLR4 binds to myeloid differentiation factor 88 (MYD88) (Yang et al., 2017; Proenca et al., 2018; Sun et al., 2019) and subsequently activates the key downstream effector nuclear factor κB (NF-κB) (Ogawa et al., 2005; Mukherji et al., 2013). NF-κB is a transcription factor that can bind to the promoter region of miR-21 and upregulate miR-21 expression, thereby resulting in the downstream target gene RASA1 being downregulated (Yang et al., 2017). RASA1 is a member of the RAS GTPase activating proteins (RAS-GAP) family and acts as a suppressor of RAS function (Donovan et al., 2002). The partial functional loss of RASA1 in miR-21 overexpressing cells can activate the GTPase activity of RAS, consequently triggering the RAS-RAF-MEK-ERK (RAS–mitogen-activated protein kinase [MAPK]) signaling pathway (Sun et al., 2013; Sun et al., 2015; Kent et al., 2016). The activation of the MAPK signaling pathway has proven to play an important part in increasing cell proliferation, eventually leading to tumorigenesis (Fang and Richardson, 2005). Taken together, miR-21 upregulation in CRC cells plays an important role in promoting colorectal carcinogenesis by *F. nucleatum via* activating the RAS-MAPK signaling pathway.

Regarding the chemotherapeutics of CRC, the combination of chemotherapeutic agents, including 5-fluorouracil (5-FU), oxaliplatin, and bevacizumab, is widely used to shrink tumor size and reduce tumor growth in advanced CRC patients (Cartwright, 2012). Although most patients with advanced CRC initially respond to combined chemotherapy, treatment outcomes may still be disappointing because of drug resistance leading to tumor recurrence, and the 5-year survival rate of patients is lower than 10% (Dahan et al., 2009). Some studies have shown that the enrichment of *F. nucleatum* is associated with recurrence postchemotherapy and shorter survival duration (Mima et al., 2016; Yu et al., 2017). In the *F. nucleatum–*mediated chemoresistance of CRC cells, miR-4802 and miR-18a^*^, which similarly depend on the TLR4 and MYD88 signaling pathways, are significantly downregulated (**Figure 2**). The selective loss of miR-4802 and miR-18a,^*^ respectively, induces the upregulation of ATG7 and ULK1, which are members of autophagy signaling elements. Therefore, when these *F. nucleatum–*infected CRC cells are treated with chemotherapy agents, such as 5-FU and oxaliplatin, the autophagy pathway will be activated and promote chemoresistance *via* suppressing cell apoptosis. Altogether, these results indicate that *F. nucleatum* may promote chemoresistance in patients with CRC *via* the selective loss of miR-4802 and miR-18a* expression and subsequent cancer autophagy activation.

### *E. coli* Promotes Cell Proliferation and Inflammation by Modulating miRNAs

**Figure 3:**
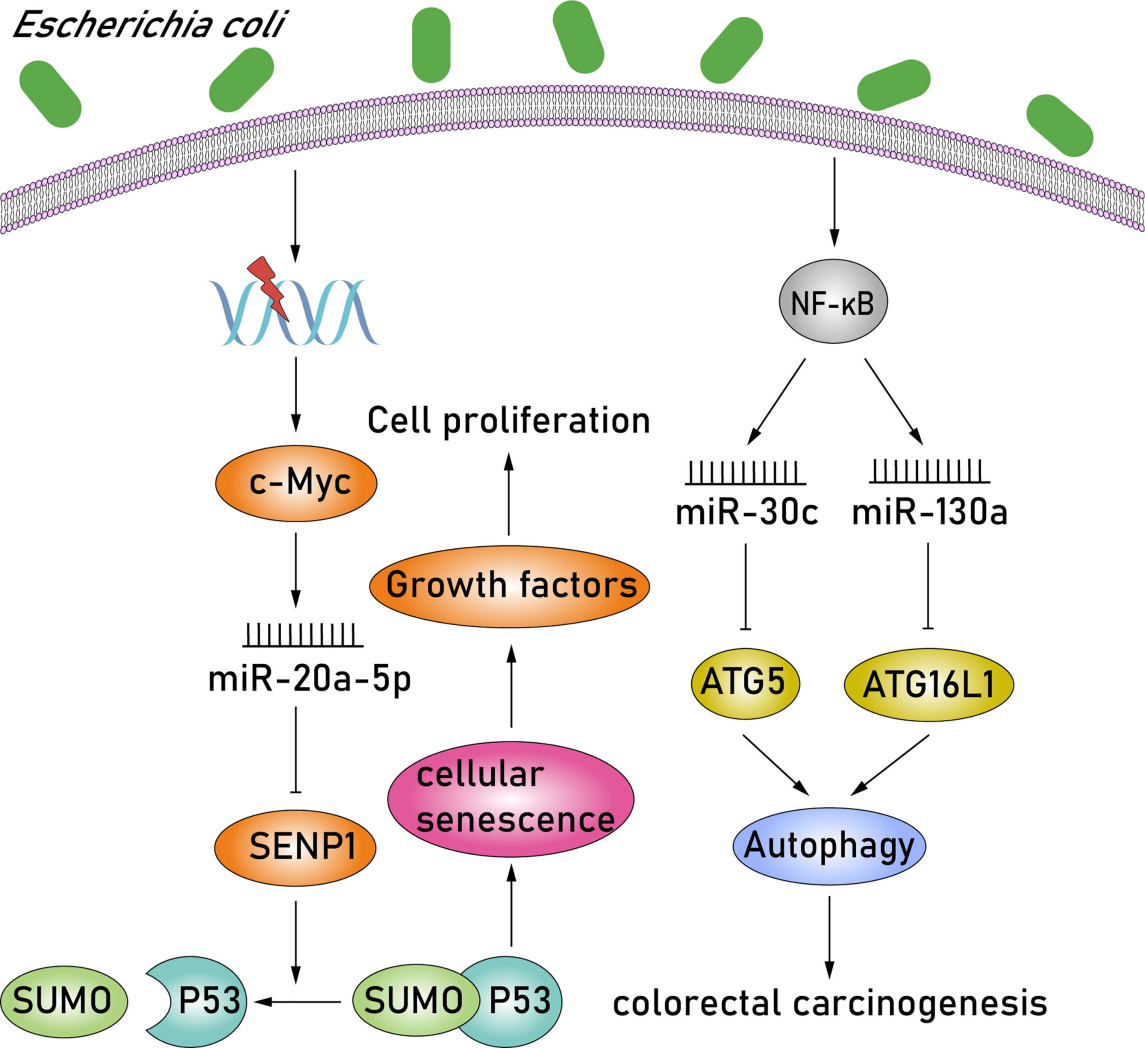
*E. coli* promotes cell proliferation and inflammation by modulating miR-20a-5p, miR-30c, and miR-130a. In *pks^+^ E. coli*–infected CRC cells, c-MYC is activated, and it subsequently results in the upregulation of miR-20a-5p. Upregulation of miR-20a-5p can cause the translational silencing of target SENP1. SENP1 is a key enzyme that blocks the modification of the SUMO1-conjugated p53 patterns. Moreover, the SUMOylation of p53 is identified as the key regulator of cellular senescence. The senescence of intestinal epithelial cells in *pks^+^ E. coli*–infected CRC cells consequently induces the secretion of growth factors, which play a crucial role in stimulating tumor growth. In addition, expressions of miR-30c and miR-130a were also upregulated significantly in AIEC-infected epithelial cells *via* activating the NF-κB pathway. Overexpression of miR-30c and miR-130a subsequently downregulates the expression of ATG5 and ATG16L1, respectively. ATG5 and ATG16L1 are members of autophagy signaling elements, and their downregulation will result in defective autophagy. Moreover, dysregulated autophagy is associated with numerous human pathologies, such as colorectal carcinogenesis.

*E. coli* is a facultative anaerobic gram-negative bacteria with pathogen-like features, such as downregulating DNA mismatch repair proteins or producing various toxins exhibiting carcinogenic features (Dalmasso et al., 2014). Certain strains of *E. coli* in group B2, containing the polyketone acid synthetase (*pks*) island, can produce the colibactin toxin, induce proproliferative effect, and are frequently associated with CRC (Buc et al., 2013; Cougnoux et al., 2014; Dalmasso et al., 2014). c-MYC, a transcription factor involved in DNA damage response, is activated in *pks^+^ E. coli*–infected CRC cells, and c-MYC subsequently binds to the miR-20a-5p promoter, resulting in the upregulation of miR-20a-5p. Upregulation of miR-20a-5p can cause the translational silencing of target SENP1 by binding to the SENP1 mRNA 3′-UTR (O'Donnell et al., 2005; Guerra et al., 2010). SENP1 is a key enzyme involved in the regulation of the SUMOylation process and blocks the modification of the small ubiquitin-like modifier 1 (SUMO1)–conjugated p53 patterns. Thus, studies have observed an accumulation of SUMO1-conjugated p53 in *pks^+^ E. coli*–infected CRC cells by downregulating SENP1. Moreover, the SUMOylation of p53 was identified as the key regulator of cellular senescence, characterized by the induction of megalocytosis and cell cycle arrest (Nougayrède et al., 2006; Yates et al., 2008). The senescence of intestinal epithelial cells in *pks^+^ E. coli*–infected CRC cells induces the secretion of growth factors, including hepatocyte growth factor, fibroblast growth factor, and granulocyte-macrophage colony-stimulating factor, which play a crucial role in stimulating tumor growth. These studies reveal a new paradigm in CRC whereby *pks^+^ E. coli*–infected CRC cells activate the c-MYC/miR-20a-5p/SENP1/senescence/growth factors pathway, consequently promoting the proliferation of uninfected cells and, in turn, stimulating tumor growth (**Figure 3**). In addition, among the miRNAs previously reported, the expression of miR-30c and miR-130a was also upregulated significantly in adherent-invasive *E. coli* (AIEC)–infected epithelial cells *via* activating the NF-κB pathway (Fasseu et al., 2010; Nguyen et al., 2014). Overexpression of miR-30c and miR-130a subsequently downregulates the expression of ATG5 and ATG16L1, respectively, by binding to the 3′-UTRs of target mRNAs. ATG5 and ATG16L1 are members of autophagy signaling elements, and their downregulation will result in defective autophagy. Autophagy is a tightly regulated homeostatic process in various physiological states, and therefore, dysregulated autophagy is associated with numerous human pathologies, such as colorectal carcinogenesis (Rubinsztein et al., 2012) (**Figure 3**). AIEC bacteria are able to invade and replicate within epithelial cells and macrophages. Studies have shown that impaired autophagy can enhance the intracellular replication of AIEC and induce the secretion of proinflammatory cytokines (Lapaquette et al., 2010; Lapaquette et al., 2012). Moreover, impaired nucleotide-binding oligomerization domain-containing protein 2 (NOD2) expression also affects bacterial autophagy and can be conducive to AIEC persistence and replication within epithelial cells and macrophages (Lapaquette et al., 2012; Nguyen et al., 2014; Negroni et al., 2016). NOD2 is a member of the nucleotide-binding oligomerization domain (NOD)–like receptors subfamily, which contains a caspase recruitment domain (CARD), and can recruit ATG16L1 to the plasma membrane at the bacterial entry site, thereby activating the autophagic response to invasive bacteria (Travassos et al., 2010; Brooks et al., 2011). Thus, NOD2 and ATG16L1 can activate an autophagy-mediated, anti-bacterial pathway, suggesting a novel method to inhibit AIEC invasion. Altogether, AIEC may increase the proinflammatory response and consequently lead to colorectal carcinogenesis *via* upregulating miR-30c and miR-130a and inducing defective autophagy, while, NOD2 and ATG16L1 may provide an approach to prevent AIEC invasion.

### Enterotoxigenic *Bacteroides fragilis* Induces Tumor Growth and Promotes Inflammation by Modulating miRNAs

**Figure 4:**
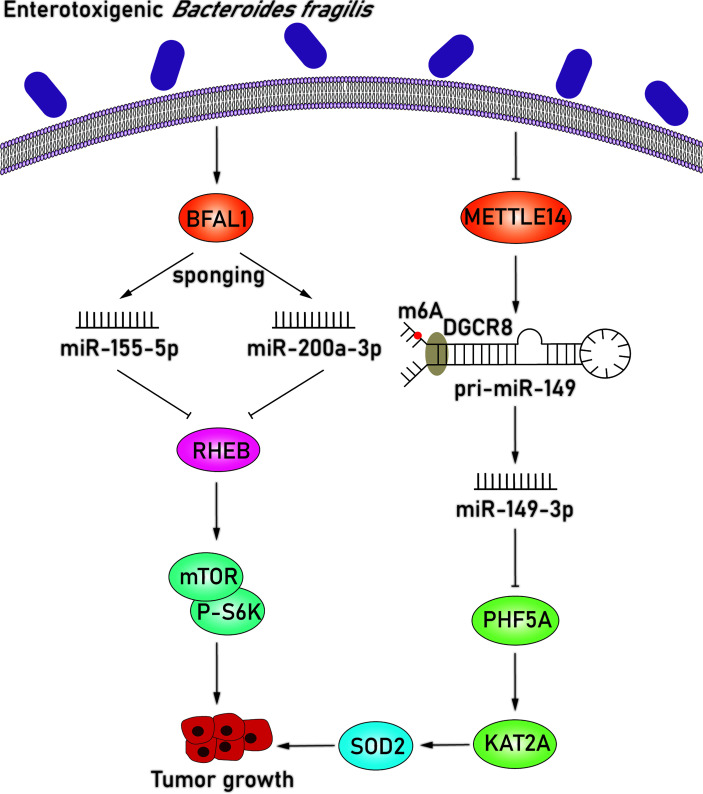
ETBF induces tumor growth by downregulating miR-155-5p, miR-200a-3p, and miR-149-3p. BFAL1 was upregulated in ETBF-infected CRC cells and subsequently decreased the levels of miR-155-5p and miR-200a-3p, which attenuated their suppressive function on target RHEB mRNA expression. The downregulated RHEB consequently activates the RHEB/mTOR pathway, thereby mediating ETBF-induced tumor growth. miR-149-3p is also significantly downregulated in ETBF-infected CRC cells. Regulation of miR-149-3p was attributed to METTL14-dependent m6A modification *via* modifying pri-miRNA splicing. In ETBF-infected CRC cells, METTL14 was downregulated so that the level of miR-149-3p was reduced, which further upregulated target PHF5A. The overexpression of PHF5A upregulated the target mRNA level of KAT2A in CRC cells, which can upregulate SOD2 *via* directly binding to the promoter region of SOD2. More importantly, the overexpression of SOD2 is relevant to poor clinical outcomes in gastric cancer, and SOD2 may promote intestinal inflammation and tumorigenesis.

Enterotoxigenic *Bacteroides fragilis* (ETBF), a subtype strain of *B. fragilis*, is one of the most prevalent species in CRC (Vétizou et al., 2015). The pathogenicity of ETBF originates mainly from the bft gene, which encodes for the *B. fragilis* toxin, and this toxin has been widely acknowledged to play a part in colorectal carcinogenesis (DeStefano Shields et al., 2016). In addition to the bacterial toxin, the mechanistic links between miRNAs and ETBF in CRC have also been explored (**Figure 4**). Studies found that *B. fragilis*–associated lncRNA1 (BFAL1) was upregulated in ETBF-infected CRC cells and confirmed that the overexpression of BFAL1 functioned as an activator of ETBF-induced CRC (Bao et al., 2019). BFAL1 is a long noncoding RNA (lncRNA) with limited coding potential; however, it acts as a regulator of numerous biological functions and pathological processes (Quinn and Chang, 2016). At the same time, overexpression of BFAL1 decreased the levels of miR-155-5p and miR-200a-3p and attenuated their suppressive function on target RHEB mRNA expression by competitively binding with target miRNAs. With the help of miRNAs, BFAL1 consequently activates the Ras homolog, which is the MTORC1 binding/mammalian target of the rapamycin (RHEB/mTOR) pathway, thereby mediating ETBF-induced tumor growth (Bao et al., 2019).

In addition, miR-149-3p, which has been proven to inhibit tumorigenesis in other cancers, was also significantly downregulated in both ETBF-infected CRC cells and exosomes derived from ETBF-infected cells (Yao and Wu, 2019). Regulation level of miR-149-3p was attributed to methyltransferase-like 14 (METTL14)–dependent N^6^-methyladenosine (m^6^A) modification *via* modifying pri-miRNA splicing (Ma et al., 2017; Cao et al., 2021). METTL14 modulates the splicing process of pri-miR-149 by recognizing the microprocessor protein DGCR8 and inducing m^6^A modification, which is a predominant internal modification of RNA in higher eukaryotes (Niu et al., 2013; Ma et al., 2017). METTL14 was downregulated in ETBF-infected CRC cells, inducing the level of miR-149-3p, which led to the upregulation of the miR-149-3p target PHD finger protein 5A (PHF5A). PHF5A plays a critical role in mRNA precursor splicing, enhances the stability of the splicing factor 3b (SF3b) complex in CRC cells, and further contributes to decreased exon skipping level. The overexpression of PHF5A subsequently upregulates the target mRNA level of KAT2A in CRC cells, which participates in the regulation of the cell cycle (Cao et al., 2021). KAT2A can regulate gene transactivation *via* H3K9ac and H3K14ac and significantly upregulate target superoxide dismutase 2 (SOD2) mitochondrial in ETBF-infected CRC cells *via* directly binding to the promoter region of SOD2 (Sun et al., 2016; Cao et al., 2021). Several studies have reported that the overexpression of SOD2 is relevant to poor clinical outcome in gastric cancer, and SOD2 may promote intestinal inflammation and tumorigenesis (Janssen et al., 2000; Chen et al., 2013). In addition to miRNA, lncRNA can similarly modify the transcription of SOD2 and promote gastric carcinogenesis, revealing a new angel in elucidating the potential mechanisms behind CRC development. Moreover, exosomes derived from ETBF-infected cells, which encapsulated downregulated miR-149-3p, can also be successfully delivered to CD4^+^ T cells and significantly reduce the overexpression of interleukin 17A, tumor necrosis factor α, and RORC, thereby resulting in an increased proinflammatory response. Similarly, these exosomes in TME can be delivered to adjacent epithelial cells and promote CRC development *via* downregulating miR-149-3p (Cao et al., 2021). Therefore, ETBF can significantly promote CRC development by regulating miR-149-3p in ETBF-infected CRC cells and by shaping the complex inflammatory TME.

### Butyrate Produced by *Faecalibacterium prausnitzii* Suppresses the Proliferation of CRC Cells by Modulating miRNAs

**Figure 5:**
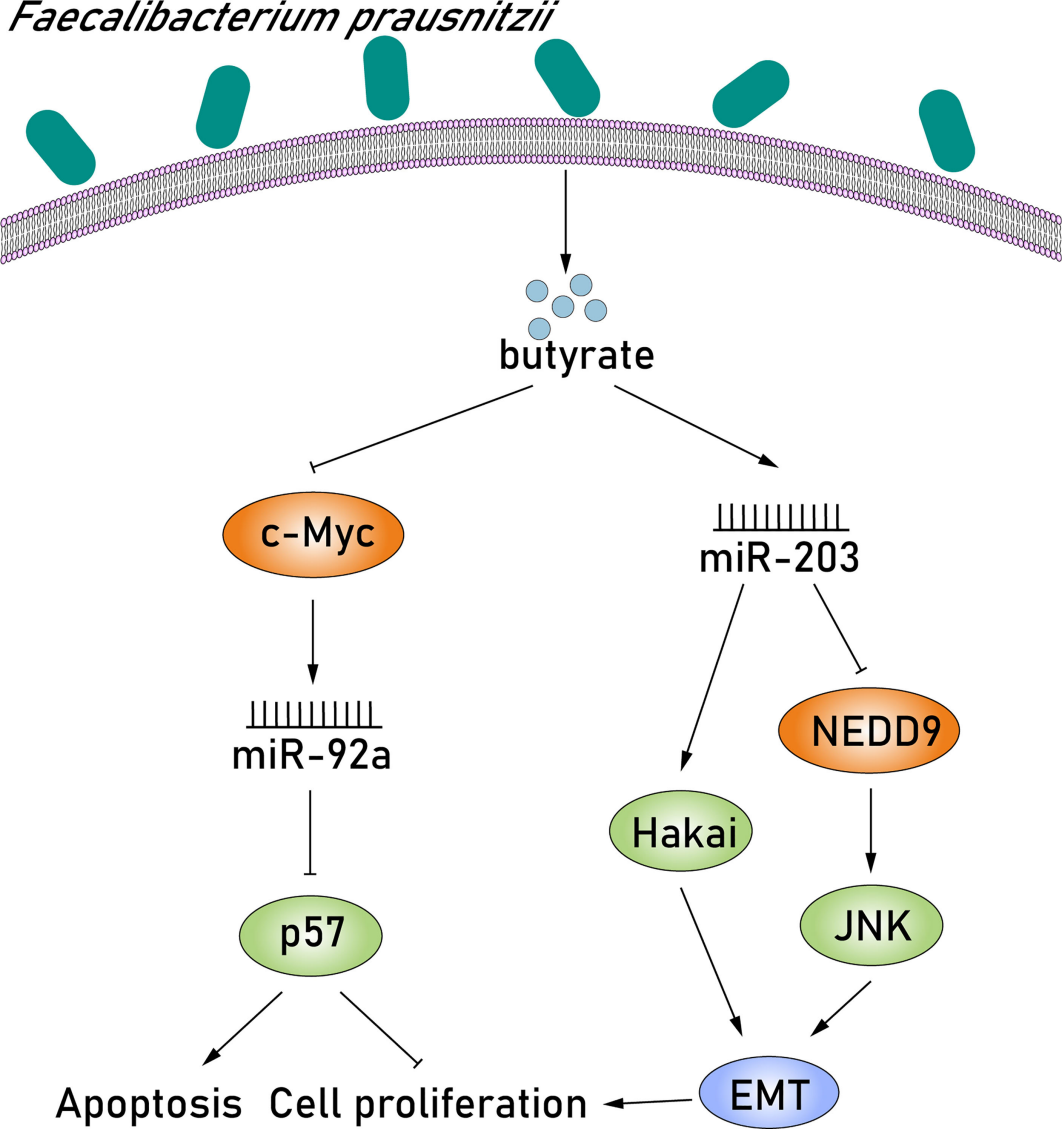
Butyrate produced by *F. prausnitzii* suppresses the proliferation of CRC cells by modulating miR-92a and miR-203. First, butyrate produced by *F. prausnitzii* suppressed oncogenic miR-92a overexpression in human CRC cells and detected a rapid decrease in the levels of c-MYC after butyrate treatment. miR-92a downregulation subsequently stimulated p57 expression, which is epigenetically silenced in cancer, blocks cell proliferation by promoting apoptosis, inhibiting angiogenesis and cell cycle arrest. In contrast, miR-203 expression is upregulated after butyrate treatment and consequently suppresses the proliferation of CRC cells *via* directly inhibiting NEDD9 expression. NEDD9, a significant tumor-promoting factor, can induce the EMT by activating JNK, thereby promoting tumor invasion and metastasis. Upregulated miR-203 induced by butyrate can also lower Hakai expression, eventually suppressing the proliferation of CRC cells.

Given the various niche metabolic pathways that the microbiome possesses, there is undoubtedly a metabolic interaction between cancer cells and the microbiome. Some of them, including short-chain fatty acids (SCFAs), vitamins, secondary bile acids, and phytochemicals, play important roles in the composition of the TME and have been found to modulate the expression of miRNAs, affecting the apoptosis, invasion, and proliferation of cancer cells (Louis et al., 2014; Johnson et al., 2016; O'Keefe, 2016). *Faecalibacterium prausnitzii* is a well-known tumor-inducing bacterium in the human gut and is considered to be an important producer of butyrate (Lenoir et al., 2020). Butyrate is the most studied SCFA, and it is synthesized by glycolysis from microbiome-accessible hydrocarbons (Louis and Flint, 2017). There is growing scientific evidence indicating that butyrate can suppress the proliferation of CRC cells and induce apoptosis and differentiation *via* dysregulating the expression of miRNAs. Hu et al. (2015) found that butyrate suppressed oncogenic miR-92a overexpression in human CRC cells and detected a rapid decrease in the levels of c-MYC and pri-miR-17-92a after butyrate treatment. Previous studies have found a highly conserved c-MYC binding site in the intronic C13orf25 promoter of the upstream pri-miR-17-92a coding sequence, suggesting that butyrate treatment reduced miR-92a levels at all processing stages (Ji et al., 2011). miR-92a downregulation subsequently stimulated p57 expression *via* reducing the inhibition of p57 translation. Butyrate-stimulated p57 protein, which is epigenetically silenced in cancer, blocks cell proliferation by promoting apoptosis, inhibiting angiogenesis and cell cycle arrest (Kavanagh and Joseph, 2011). Thus, butyrate, produced by *F. prausnitzii*, decreases oncogenic miR-92a levels by suppressing c-MYC protein levels, thereby activating p57 translation and inhibiting CRC proliferation (**Figure 5**). In contrast, miR-203 expression is upregulated after butyrate treatment and consequently suppresses the proliferation of CRC cells *via* directly inhibiting NEDD9 expression. NEDD9, a significant tumor-promoting factor, is a member of the crk-associated substrate family and was found highly expressed in CRC tissues (Han et al., 2016). Studies have demonstrated that NEDD9 can induce the epithelial–mesenchymal transition (EMT) by activating c-Jun NH-terminal kinase (JNK), thereby promoting tumor invasion and metastasis (Meng et al., 2019). In this pathway, EMT is a reversible process of differentiation that results in the absence of E-calcium adhesion protein (the main ingredient of adhesion) in epithelial cells and JNK, a member of the MAPK family, which has been reported to be closely associated with proliferation, differentiation, apoptosis, and migration (Wagner and Nebreda, 2009; Min et al., 2015). Notably, it has been extensively studied that EMT is characterized by the loss of E-cadherin in epithelial cells, resulting in the downregulation of cell–cell adhesion, suggesting the great impact of E-cadherin inactivation in colorectal carcinogenesis (Meng et al., 2019). Hakai is the first posttranslational regulator described for the E-cadherin stability and has been reported to cause the alteration of cell–cell contacts involved in colorectal carcinogenesis (Castosa et al., 2018). Upregulated miR-203 induced by butyrate can also lower Hakai expression by binding to the 3′-UTR of target mRNA, eventually suppressing the proliferation of CRC cells (Abella et al., 2012) (**Figure 5**). Taken together, butyrate produced by *F. prausnitzii* can suppress the proliferation of CRC cells by upregulating miR-203, which can not only inhibit NEDD9 expression, but also inhibit Hakai expression.

## Conclusion

Modulation of miRNAs by the microbiome during bacterial pathogen infection and its effects on the host transcriptome have been investigated. Given the role of miRNAs in affecting many critical cellular processes, such as apoptosis, proliferation, and differentiation, we believe that miRNAs play a central, if not critical, role in influencing the development of CRC. In order to fully understand the interactions between unique miRNAs and microbiome, we investigated several possible pairwise interactions in this article and elaborated on the mechanisms involved. The four pathogens *F. nucleatum*, *E. coli*, ETBF, and *F. prausnitzii* are introduced; however, the interactions between other pathogens and CRC-related miRNA still need to be studied. As miRNAs have an important role in CRC, regulating miRNAs for therapeutic interventions is needed. Of note, regulating the composition of the gut microbiome and reducing the occurrence of CRC, such as fecal microorganism transplantation, need to be applied. In the future, it will be imperative to use a combination of approaches to comprehensively treat CRC to effectively reduce the recurrence of CRC.

## Author Contributions

JX and YL have contributed equally to this work and share first authorship. The study was conceptualized by JX and YL. HZ and ZZ were responsible for literature search. JX and YL were responsible for writing of the original draft preparation. DT and DW were responsible for writing, review, and editing. Elaboration of the tables and figures was performed by JZ and WZ. JX and YL were responsible for Supervision. All authors have read and agreed to the published version of the manuscript.

## Funding

This study was supported by Training Project of Key Talents of Youth Medicine in Jiangsu province, China [No. QNRC2016330], The Key Disease Standardization Diagnosis and Treatment Project in Jiangsu Province [NO. BE2015664], The Academic Science and Technology Innovation Fund for College Students [No. ×20180714], The Social Development-Health Care Project of Yangzhou, Jiangsu Province [No. YZ2018087], The Social Development Project of Yangzhou, Jiangsu Province [No. YZ2021075], The Graduate Research and Practice Innovation Plan of Graduate Education Innovation Project in Jiagsu Province [NO. SJCX211644] and High-level talent “six one project” top talent scientific project of jiangsu Province [No. LGY2019034].

## Conflict of Interest

The authors declare that the research was conducted in the absence of any commercial or financial relationships that could be construed as a potential conflict of interest.

## Publisher’s Note

All claims expressed in this article are solely those of the authors and do not necessarily represent those of their affiliated organizations, or those of the publisher, the editors and the reviewers. Any product that may be evaluated in this article, or claim that may be made by its manufacturer, is not guaranteed or endorsed by the publisher.

## References

[B1] Abella V. Valladares M. Rodriguez T. Haz M. Blanco M. Tarrio N. (2012). miR-203 Regulates Cell Proliferation Through Its Influence on Hakai Expression. PloS One 7 (12), e52568. doi: 10.1371/journal.pone.0052568 23285092 PMC3527564

[B2] Archambaud C. Sismeiro O. Toedling J. Soubigou G. Bécavin C. Lechat P. (2013). The Intestinal Microbiota Interferes With the microRNA Response Upon Oral Listeria Infection. mBio. 4, 6. doi: 10.1128/mBio.00707-13 PMC3870255 24327339

[B3] Baek D. Villén J. Shin C. Camargo F. Gygi S. Bartel D. (2008). The Impact of microRNAs on Protein Output. Nature 455, 7209, 64–71. doi: 10.1038/nature07242 18668037 PMC2745094

[B4] Bao Y. Tang J. Qian Y. Sun T. Chen H. Chen Z. (2019). Long Noncoding RNA BFAL1 Mediates Enterotoxigenic Bacteroides fragilis-Related Carcinogenesis in Colorectal Cancer *via* the RHEB/mTOR Pathway. Cell Death Dis. 10 (9), 675. doi: 10.1038/s41419-019-1925-2 31515468 PMC6742644

[B5] Bartel D. J. C. (2004). MicroRNAs: Genomics, Biogenesis, Mechanism, and Function. Cell 116, 2, 281–297. doi: 10.1016/s0092-8674(04)00045-5 14744438

[B6] Borchert G. Lanier W. Davidson B. J. N. (2006). RNA Polymerase III Transcribes Human microRNAs. Nat Struct Mol Biol. 13, 12, 1097–1101. doi: 10.1038/nsmb1167 17099701

[B7] Bordonaro M. Lazarova D. (2015). CREB-Binding Protein, P300, Butyrate, and Wnt Signaling in Colorectal Cancer. World J. Gastroenterol. 1 (27), 8238–8248. doi: 10.3748/wjg.v21.i27.8238 PMC4507093 26217075

[B8] Brooks M. Rajaram M. Azad A. Amer A. Valdivia-Arenas M. Park J. (2011). NOD2 Controls the Nature of the Inflammatory Response and Subsequent Fate of Mycobacterium tuberculosis and M. bovis BCG in Human Macrophages. Cell Microbiol. 13, 3, 402–418. doi: 10.1111/j.1462-5822.2010.01544.x 21040358 PMC3259431

[B9] Buc E. Dubois D. Sauvanet P. Raisch J. Delmas J. Darfeuille-Michaud A. (2013). High Prevalence of Mucosa-Associated E. coli Producing Cyclomodulin and Genotoxin in Colon Cancer. PLoS One 8 (2), e56964. doi: 10.1371/journal.pone.0056964 23457644 PMC3572998

[B10] Bullock M. Pickard K. Nielsen B. Sayan A. Jenei V. Mellone M. (2013). Pleiotropic Actions of miR-21 Highlight the Critical Role of Deregulated Stromal microRNAs During Colorectal Cancer Progression. Cell Death Dis. 4. doi: 10.1038/cddis.2013.213 PMC3702298 23788041

[B11] Cao Y. Wang Z. Yan Y. Ji L. He J. Xuan B. (2021). Enterotoxigenic Bacteroides fragilis Promotes Intestinal Inflammation and Malignancy by Inhibiting Exosomes-Packaged miR-149-3p. Gastroenterology 161 (5), 1552–1566. doi: 10.1053/j.gastro.2021.08.003 34371001

[B12] Cartwright T. (2012). Treatment Decisions After Diagnosis of Metastatic Colorectal Cancer. Clin. Colorectal Cancer 11, 3, 155–166. doi: 10.1016/j.clcc.2011.11.001 22192364

[B13] Castosa R. Martinez-Iglesias O. Roca-Lema D. Casas-Pais A. Diaz-Diaz A. Iglesias P. (2018). Hakai Overexpression Effectively Induces Tumour Progression and Metastasis *In Vivo* . Sci. Rep. 8 (1), 3466. doi: 10.1038/s41598-018-21808-w 29472634 PMC5823865

[B14] Chen X. Huang Q. Yang S. Chu Y. Yan Y. Han L. (2015). Role of Micro RNAs in the Pathogenesis of Rheumatoid Arthritis: Novel Perspectives Based on Review of the Literature. Medicine (Baltimore) 94, 31. doi: 10.1097/md.0000000000001326 PMC4616618 26252320

[B15] Chen J. Papp G. Szodoray P. Zeher M. J. (2016). The Role of microRNAs in the Pathogenesis of Autoimmune Diseases. Autoimmun. Rev. 15 (12), 1171–1180. doi: 10.1016/j.autrev.2016.09.003 27639156

[B16] Chen P. M. Wu T. C. Wang Y. C. Cheng Y. W. Sheu G. T. Chen C. Y. (2013). Activation of NF-κB by SOD2 Promotes the Aggressiveness of Lung Adenocarcinoma by Modulating NKX2-1-Mediated IKKβ Expression. Carcinogenesis 34 (11), 2655–2663. doi: 10.1093/carcin/bgt220 23784082

[B17] Cougnoux A. Dalmasso G. Martinez R. Buc E. Delmas J. Gibold L. (2014). Bacterial Genotoxin Colibactin Promotes Colon Tumour Growth by Inducing a Senescence-Associated Secretory Phenotype. Gut 63 (12), 1932–1942. doi: 10.1136/gutjnl-2013-305257 24658599

[B18] Dahan L. Sadok A. Formento J. Seitz J. Kovacic H. (2009). Modulation of Cellular Redox State Underlies Antagonism Between Oxaliplatin and Cetuximab in Human Colorectal Cancer Cell Lines. Br. J. Pharmacol. 158, 2, 610–620. doi: 10.1111/j.1476-5381.2009.00341.x 19732064 PMC2757701

[B19] Dalmasso G. Cougnoux A. Delmas J. Darfeuille-Michaud A. Bonnet R. (2014). The Bacterial Genotoxin Colibactin Promotes Colon Tumor Growth by Modifying the Tumor Microenvironment. Gut Microbes 5 (5), 675–680. doi: 10.4161/19490976.2014.969989 25483338 PMC4615906

[B20] Dalmasso G. Nguyen H. Yan Y. Laroui H. Charania M. Ayyadurai S. (2011). Microbiota Modulate Host Gene Expression *via* microRNAs. PLoS One 6, 4. doi: 10.1371/journal.pone.0019293 PMC3084815 21559394

[B21] DeStefano Shields C. Van Meerbeke S. Housseau F. Wang H. Huso D. Casero R. (2016). Reduction of Murine Colon Tumorigenesis Driven by Enterotoxigenic Bacteroides fragilis Using Cefoxitin Treatment. J. Infect. Dis. 214, 1, 122–129. doi: 10.1093/infdis/jiw069 26908749 PMC4907405

[B22] Donovan S. Shannon K. Bollag G. (2002). GTPase Activating Proteins: Critical Regulators of Intracellular Signaling. Biochim. Biophys. Acta 1602, 1, 23–45. doi: 10.1016/s0304-419x(01)00041-5 11960693

[B23] Fabian M. Sonenberg N. (2012). The Mechanics of miRNA-Mediated Gene Silencing: A Look Under the Hood of miRISC. Nat. Struct. Mol. Biol. 19 (6), 586–593. doi: 10.1038/nsmb.2296 22664986

[B24] Fang J. Richardson B. (2005). The MAPK Signalling Pathways and Colorectal Cancer. Lancet Oncol. 6 (5), 322–327. doi: 10.1016/s1470-2045(05)70168-6 15863380

[B25] Fanini F. Fabbri M. (2017). Cancer-Derived Exosomic microRNAs Shape the Immune System Within the Tumor Microenvironment: State of the Art. Semin. Cell Dev. Biol. 67, 23–28. doi: 10.1016/j.semcdb.2016.12.004 27956165 PMC5466544

[B26] Fasseu M. Tréton X. Guichard C. Pedruzzi E. Cazals-Hatem D. Richard C. (2010). Identification of Restricted Subsets of Mature microRNA Abnormally Expressed in Inactive Colonic Mucosa of Patients With Inflammatory Bowel Disease. PLoS One 5 (10), e13160. doi: 10.1371/journal.pone.0013160 20957151 PMC2950152

[B27] Feng Y. Y. Zeng D. Z. Tong Y. N. Lu X. X. Dun G. D. Tang B. (2019). Alteration of microRNA-4474/4717 Expression and CREB-Binding Protein in Human Colorectal Cancer Tissues Infected With Fusobacterium nucleatum. PloS One 14 (4), e0215088. doi: 10.1371/journal.pone.0215088 30951563 PMC6450631

[B28] Fukugaiti M. Ignacio A. Fernandes M. Ribeiro Júnior U. Nakano V. Avila-Campos M. J. (2015). High Occurrence of Fusobacterium nucleatum and Clostridium Difficile in the Intestinal Microbiota of Colorectal Carcinoma Patients. Braz. J. Microbiol. 46 (4), 1135–1140. doi: 10.1590/s1517-838246420140665 26691472 PMC4704648

[B29] Guerra L. Albihn A. Tronnersjö S. Yan Q. Guidi R. Stenerlöw B. (2010). Myc is Required for Activation of the ATM-Dependent Checkpoints in Response to DNA Damage. PLoS One 5, 1. doi: 10.1371/journal.pone.0008924 PMC2811743 20111719

[B30] Han J. Lee Y. Yeom K. Kim Y. Jin H. Kim V. J. G. (2004). The Drosha-DGCR8 Complex in Primary microRNA Processing. Genes Dev. 18, 24, 3016–3027. doi: 10.1101/gad.1262504 15574589 PMC535913

[B31] Han R. Sun Q. Wu J. Zheng P. Zhao G. (2016). Sodium Butyrate Upregulates miR-203 Expression to Exert Anti-Proliferation Effect on Colorectal Cancer Cells. Cell Physiol. Biochem. 39 (5), 1919–1929. doi: 10.1159/000447889 27771718

[B32] Hu S. Liu L. Chang E. B. Wang J. Y. Raufman J. P. (2015). Butyrate Inhibits Pro-Proliferative miR-92a by Diminishing C-Myc-Induced miR-17-92a Cluster Transcription in Human Colon Cancer Cells. Mol. Cancer 14, 180. doi: 10.1186/s12943-015-0450-x 26463716 PMC4604099

[B33] Janssen A. M. Bosman C. B. van Duijn W. Oostendorp-van de Ruit M. M. Kubben F. J. Griffioen G. (2000). Superoxide Dismutases in Gastric and Esophageal Cancer and the Prognostic Impact in Gastric Cancer. Clin. Cancer Res. 6 (8), 3183–3192. doi: 10.1093/carcin/21.8.1623 10955802

[B34] Ji M. Rao E. Ramachandrareddy H. Shen Y. Jiang C. Chen J. (2011). The miR-17-92 microRNA Cluster is Regulated by Multiple Mechanisms in B-Cell Malignancies. Am. J. Pathol. 179, 4, 1645–1656. doi: 10.1016/j.ajpath.2011.06.008 21806958 PMC3181398

[B35] Johnson C. Spilker M. Goetz L. Peterson S. Siuzdak G. (2016). Metabolite and Microbiome Interplay in Cancer Immunotherapy. Cancer Res. 76 (21), 6146–6152. doi: 10.1158/0008-5472.Can-16-0309 27729325 PMC5093024

[B36] Kavanagh E. Joseph B. (2011). The Hallmarks of CDKN1C (P57, KIP2) in Cancer. Biochim. Biophys. Acta 1816 (1), 50–56. doi: 10.1016/j.bbcan.2011.03.002 21447370

[B37] Keller S. Ridinger J. Rupp A. Janssen J. Altevogt P. (2011). Body Fluid Derived Exosomes as a Novel Template for Clinical Diagnostics. J. Transl. Med. 9, 86. doi: 10.1186/1479-5876-9-86 21651777 PMC3118335

[B38] Kent O. Mendell J. Rottapel R. (2016). Transcriptional Regulation of miR-31 by Oncogenic KRAS Mediates Metastatic Phenotypes by Repressing RASA1. Mol. Cancer Res. 14 (3), 267–277. doi: 10.1158/1541-7786.Mcr-15-0456 26747707 PMC4794362

[B39] Kim J. Wieckowski E. Taylor D. Reichert T. Watkins S. Whiteside T. L. (2005). Fas Ligand-Positive Membranous Vesicles Isolated From Sera of Patients With Oral Cancer Induce Apoptosis of Activated T Lymphocytes. Clin. Cancer Res. 11 (3), 1010–1020. doi: 10.1158/1078-0432.1010.11.3 15709166

[B40] Kohlhapp F. Mitra A. Lengyel E. Peter M. (2015). MicroRNAs as Mediators and Communicators Between Cancer Cells and the Tumor Microenvironment. Oncogene 34 (48), 5857–5868. doi: 10.1038/onc.2015.89 25867073 PMC4604012

[B41] Kumar M. Lu J. Mercer K. Golub T. Jacks T. (2007). Impaired microRNA Processing Enhances Cellular Transformation and Tumorigenesis. Nat. Genet. 39 (5), 673–677. doi: 10.1038/ng2003 17401365

[B42] Lapaquette P. Bringer M. Darfeuille-Michaud A. (2012). Defects in Autophagy Favour Adherent-Invasive Escherichia coli Persistence Within Macrophages Leading to Increased Pro-Inflammatory Response. Cell Microbiol. 14 (6), 791–807. doi: 10.1111/j.1462-5822.2012.01768.x 22309232

[B43] Lapaquette P. Glasser A. Huett A. Xavier R. Darfeuille-Michaud A. (2010). Crohn's Disease-Associated Adherent-Invasive E. coli Are Selectively Favoured by Impaired Autophagy to Replicate Intracellularly. Cell Microbiol. 12, 1, 99–113. doi: 10.1111/j.1462-5822.2009.01381.x 19747213 PMC3743084

[B44] Lee Y. Kim M. Han J. Yeom K. Lee S. Baek S. (2004). MicroRNA Genes Are Transcribed by RNA Polymerase II. EMBO J. 23, 20, 4051–4060. doi: 10.1038/sj.emboj.7600385 15372072 PMC524334

[B45] Lenoir M. Martin R. Torres-Maravilla E. Chadi S. Gonzalez-Davila P. Sokol H. (2020). Butyrate Mediates Anti-Inflammatory Effects of Faecalibacterium prausnitzii in Intestinal Epithelial Cells Through Dact3. Gut Microbes 12 (1), 1–16. doi: 10.1080/19490976.2020.1826748 PMC7567499 33054518

[B46] Li Y. Lauriola M. Kim D. Francesconi M. D'Uva G. Shibata D. (2016). Adenomatous Polyposis Coli (APC) Regulates Mir17-92 Cluster Through β-Catenin Pathway in Colorectal Cancer. Oncogene 35, 35, 4558–4568. doi: 10.1038/onc.2015.522 26804172 PMC4960006

[B47] Li L. Sarver A. Khatri R. Hajeri P. Kamenev I. French A. (2014). Sequential Expression of miR-182 and miR-503 Cooperatively Targets FBXW7, Contributing to the Malignant Transformation of Colon Adenoma to Adenocarcinoma. J. Pathol. 234 (4), 488–501. doi: 10.1002/path.4407 25269767

[B48] Liu S. da Cunha A. Rezende R. Cialic R. Wei Z. Bry L. (2016). The Host Shapes the Gut Microbiota *via* Fecal MicroRNA. Cell Host Microbe 19, 1, 32–43. doi: 10.1016/j.chom.2015.12.005 26764595 PMC4847146

[B49] Louis P. Flint H. J. (2017). Formation of Propionate and Butyrate by the Human Colonic Microbiota. Environ. Microbiol. 19 (1), 29–41. doi: 10.1111/1462-2920.13589 27928878

[B50] Louis P. Hold G. Flint H. J. (2014). The Gut Microbiota, Bacterial Metabolites and Colorectal Cancer. Nat. Rev. Microbiol. 12 (10), 661–672. doi: 10.1038/nrmicro3344 25198138

[B51] Ma J. Yang F. Zhou C. Liu F. Yuan J. Wang F. (2017). METTL14 Suppresses the Metastatic Potential of Hepatocellular Carcinoma by Modulating N -Methyladenosine-Dependent Primary MicroRNA Processing. Hepatology 65 (2), 529–543. doi: 10.1002/hep.28885 27774652

[B52] Meng H. Wu J. Huang Q. Yang X. Yang K. Qiu Y. (2019). NEDD9 Promotes Invasion and Migration of Colorectal Cancer Cell Line HCT116 *via* JNK/EMT. Oncol. Lett. 18, 4, 4022–4029. doi: 10.3892/ol.2019.10756 31516604 PMC6732989

[B53] Mima K. Nishihara R. Qian Z. Cao Y. Sukawa Y. Nowak J. (2016). Fusobacterium nucleatum in Colorectal Carcinoma Tissue and Patient Prognosis. Gut 65, 12, 1973–1980. doi: 10.1136/gutjnl-2015-310101 26311717 PMC4769120

[B54] Min J. Liu L. Li X. Jiang J. Wang J. Zhang B. (2015). Absence of DAB2IP Promotes Cancer Stem Cell Like Signatures and Indicates Poor Survival Outcome in Colorectal Cancer. Sci. Rep. 5, 16578. doi: 10.1038/srep16578 26564738 PMC4643237

[B55] Mukherji A. Kobiita A. Ye T. Chambon P. (2013). Homeostasis in Intestinal Epithelium Is Orchestrated by the Circadian Clock and Microbiota Cues Transduced by TLRs. Cell 153, 4, 812–827. doi: 10.1016/j.cell.2013.04.020 23663780

[B56] Negroni A. Colantoni E. Vitali R. Palone F. Pierdomenico M. Costanzo M. (2016). NOD2 Induces Autophagy to Control AIEC Bacteria Infectiveness in Intestinal Epithelial Cells. Inflamm. Res. 65, 10, 803–813. doi: 10.1007/s00011-016-0964-8 27335178

[B57] Nguyen H. T. Dalmasso G. Muller S. Carriere J. Seibold F. Darfeuille-Michaud A. (2014). Crohn's Disease-Associated Adherent Invasive Escherichia coli Modulate Levels of microRNAs in Intestinal Epithelial Cells to Reduce Autophagy. Gastroenterology 146 (2), 508–519. doi: 10.1053/j.gastro.2013.10.021 24148619

[B58] Niu Y. Zhao X. Wu Y. Li M. Wang X. Yang Y. G. (2013). N6-Methyl-Adenosine (M6a) in RNA: An Old Modification With a Novel Epigenetic Function. Genomics Proteomics Bioinformatics 11 (1), 8–17. doi: 10.1016/j.gpb.2012.12.002 23453015 PMC4357660

[B59] Nougayrède J. Homburg S. Taieb F. Boury M. Brzuszkiewicz E. Gottschalk G. (2006). Escherichia coli Induces DNA Double-Strand Breaks in Eukaryotic Cells. Science 313, 5788, 848–851. doi: 10.1126/science.1127059 16902142

[B60] O'Donnell K. Wentzel E. Zeller K. Dang C. Mendell J. (2005). C-Myc-Regulated microRNAs Modulate E2F1 Expression. Nature 435, 7043, 839–843. doi: 10.1038/nature03677 15944709

[B61] O'Keefe S. J. (2016). Diet, Microorganisms and Their Metabolites, and Colon Cancer. Nat. Rev. Gastroenterol. Hepatol. 13 (12), 691–706. doi: 10.1038/nrgastro.2016.165 27848961 PMC6312102

[B62] Ogawa S. Lozach J. Benner C. Pascual G. Tangirala R. Westin S. (2005). Molecular Determinants of Crosstalk Between Nuclear Receptors and Toll-Like Receptors. Cell 122, 5, 707–721. doi: 10.1016/j.cell.2005.06.029 16143103 PMC1430687

[B63] Proenca M. A. Biselli J. M. Succi M. Severino F. E. Berardinelli G. N. Caetano A. (2018). Relationship Between Fusobacterium nucleatum, Inflammatory Mediators and microRNAs in Colorectal Carcinogenesis. World J. Gastroenterol. 24 (47), 5351–5365. doi: 10.3748/wjg.v24.i47.5351 30598580 PMC6305535

[B64] Quinn J. Chang H. (2016). Unique Features of Long Non-Coding RNA Biogenesis and Function. Nat. Rev. Genet. 17 (1), 47–62. doi: 10.1038/nrg.2015.10 26666209

[B65] Raposo G. Stoorvogel W. (2013). Extracellular Vesicles: Exosomes, Microvesicles, and Friends. J. Cell Biol. 200 (4), 373–383. doi: 10.1083/jcb.201211138 23420871 PMC3575529

[B66] Rubinsztein D. Codogno P. Levine B. (2012). Autophagy Modulation as a Potential Therapeutic Target for Diverse Diseases. Nat. Rev. Drug Discov. 11 (9), 709–730. doi: 10.1038/nrd3802 22935804 PMC3518431

[B67] Shi C. Yang Y. Xia Y. Okugawa Y. Yang J. Liang Y. (2016). Novel Evidence for an Oncogenic Role of microRNA-21 in Colitis-Associated Colorectal Cancer. Gut 65, 9, 1470–1481. doi: 10.1136/gutjnl-2014-308455 25994220

[B68] Slattery M. Mullany L. Sakoda L. Samowitz W. Wolff R. Stevens J. (2018). Expression of Wnt-Signaling Pathway Genes and Their Associations With miRNAs in Colorectal Cancer. Oncotarget 9, 5, 6075–6085. doi: 10.18632/oncotarget.23636 29464056 PMC5814196

[B69] Sun T. He J. Liang Q. Ren L. Yan T. Yu T. (2016). LncRNA GClnc1 Promotes Gastric Carcinogenesis and May Act as a Modular Scaffold of WDR5 and KAT2A Complexes to Specify the Histone Modification Pattern. Cancer Discov. 6 (7), 784–801. doi: 10.1158/2159-8290.Cd-15-0921 27147598

[B70] Sun C. H. Li B. B. Wang B. Zhao J. Zhang X. Y. Li T. T. (2019). The Role of Fusobacterium nucleatum in Colorectal Cancer: From Carcinogenesis to Clinical Management. Chronic Dis. Transl. Med. 5 (3), 178–187. doi: 10.1016/j.cdtm.2019.09.001 31891129 PMC6926109

[B71] Sun D. Wang C. Long S. Ma Y. Guo Y. Huang Z. (2015). C/EBP-β-Activated microRNA-223 Promotes Tumour Growth Through Targeting RASA1 in Human Colorectal Cancer. Br. J. Cancer 112 (9), 1491–1500. doi: 10.1038/bjc.2015.107 25867276 PMC4453668

[B72] Sun D. Yu F. Ma Y. Zhao R. Chen X. Zhu J. (2013). MicroRNA-31 Activates the RAS Pathway and Functions as an Oncogenic MicroRNA in Human Colorectal Cancer by Repressing RAS P21 GTPase Activating Protein 1 (RASA1). J. Biol. Chem. 288 (13), 9508–9518. doi: 10.1074/jbc.M112.367763 23322774 PMC3611019

[B73] Travassos L. H. Carneiro L. A. Ramjeet M. Hussey S. Kim Y. G. Magalhães J. G. (2010). Nod1 and Nod2 Direct Autophagy by Recruiting ATG16L1 to the Plasma Membrane at the Site of Bacterial Entry. Nat. Immunol. 11 (1), 55–62. doi: 10.1038/ni.1823 19898471

[B74] Vétizou M. Pitt J. Daillère R. Lepage P. Waldschmitt N. Flament C. (2015). Anticancer Immunotherapy by CTLA-4 Blockade Relies on the Gut Microbiota. Science 350 (6264), 1079–1084. doi: 10.1126/science.aad1329 26541610 PMC4721659

[B75] Wagner E. Nebreda A. (2009). Signal Integration by JNK and P38 MAPK Pathways in Cancer Development. Nat. Rev. Cancer 9 (8), 537–549. doi: 10.1038/nrc2694 19629069

[B76] Wang T. Cai G. Qiu Y. Fei N. Zhang M. Pang X. (2012). Structural Segregation of Gut Microbiota Between Colorectal Cancer Patients and Healthy Volunteers. ISME J. 6 (2), 320–329. doi: 10.1038/ismej.2011.109 21850056 PMC3260502

[B77] Winter J. Diederichs S. (2011). MicroRNA Biogenesis and Cancer. Methods Mol. Biol. 676, 3–22. doi: 10.1007/978-1-60761-863-8_1 20931386

[B78] Winter J. Jung S. Keller S. Gregory R. Diederichs S. (2009). Many Roads to Maturity: microRNA Biogenesis Pathways and Their Regulation. Nat. Cell Biol. 11 (3), 228–234. doi: 10.1038/ncb0309-228 19255566

[B79] Yang Y. Weng W. Peng J. Hong L. Yang L. Toiyama Y. (2017). Fusobacterium nucleatum Increases Proliferation of Colorectal Cancer Cells and Tumor Development in Mice by Activating Toll-Like Receptor 4 Signaling to Nuclear Factor-KappaB, and Up-Regulating Expression of MicroRNA-21. Gastroenterology 152 (4), 851–866.e824. doi: 10.1053/j.gastro.2016.11.018 27876571 PMC5555435

[B80] Yao J. Wu X. (2019). Upregulation Of miR-149-3p Suppresses Spinal Chordoma Malignancy By Targeting Smad3. Onco. Targerts Ther. 12, 9987–9997. doi: 10.2147/ott.S222380 PMC6875263 31819495

[B81] Yates K. Korbel G. Shtutman M. Roninson I. DiMaio D. (2008). Repression of the SUMO-Specific Protease Senp1 Induces P53-Dependent Premature Senescence in Normal Human Fibroblasts. Aging Cell 7 (5), 609–621. doi: 10.1111/j.1474-9726.2008.00411.x 18616636 PMC2745089

[B82] Yi R. Li Y. Wang F. L. Miao G. Qi R. M. Zhao Y. Y. (2016). MicroRNAs as Diagnostic and Prognostic Biomarkers in Colorectal Cancer. World J. Gastrointest. Oncol. 8 (4), 330–340. doi: 10.4251/wjgo.v8.i4.330 27096028 PMC4824711

[B83] Yi R. Qin Y. Macara I. Cullen B. (2003). Exportin-5 Mediates the Nuclear Export of pre-microRNAs and Short Hairpin RNAs. Genes Dev. 17 (24), 3011–3016. doi: 10.1101/gad.1158803 14681208 PMC305252

[B84] Yuan C. Burns M. B. Subramanian S. Blekhman R. (2018). Interaction Between Host MicroRNAs and the Gut Microbiota in Colorectal Cancer. mSystems 3 (3), e00205–17. doi: 10.1128/mSystems.00205-17 PMC5954203 29795787

[B85] Yu T. Guo F. Yu Y. Sun T. Ma D. Han J. (2017). Fusobacterium nucleatum Promotes Chemoresistance to Colorectal Cancer by Modulating Autophagy. Cell 170 (3), 548–563.e516. doi: 10.1016/j.cell.2017.07.008 28753429 PMC5767127

